# Case Report: Conservative management of pediatric thyroid abscess with multispatial extension and transient thyrotoxicosis

**DOI:** 10.3389/fped.2026.1951613

**Published:** 2026-10-20

**Authors:** Abdullah K. Almutairi

**Affiliations:** 1 Department of Pediatrics, College of Medicine, Imam Abdulrahman Bin Faisal University, Dammam, Saudi Arabia; 2 Department of Pediatrics, Department of Pediatrics, King Fahd Hospital of the University, Al-Khobar, Saudi Arabia

**Keywords:** acute suppurative thyroiditis, conservative management, deep neck space infection, pediatric, pyriform sinus fistula, thyroid abscess, transient thyrotoxicosis

## Abstract

Acute suppurative thyroiditis (AST) is a rare, potentially life-threatening bacterial infection of the thyroid gland, and surgical drainage has traditionally been regarded as standard care when an abscess forms. We report a case of a previously healthy 9-year-old boy who presented with fever, odynophagia, hoarseness, and neck stiffness and was found to have a left thyroid lobe abscess with multispatial cervical extension into the visceral, prethyroid, retropharyngeal, and parapharyngeal spaces, accompanied by transient biochemical thyrotoxicosis. Because the airway remained patent and systemic toxicity was absent, the patient was managed conservatively with intravenous antibiotics despite a non-yielding ultrasound-guided aspiration, resulting in rapid clinical improvement and normalization of inflammatory markers without operative drainage or recurrence at follow-up. Flexible laryngoscopy and a barium swallow showed no evidence of a pyriform sinus fistula, and thyroid function normalized without antithyroid therapy. This case highlights that carefully selected children with thyroid abscesses may recover without surgery despite radiological multispatial extension, and that the transient thyrotoxicosis of AST reflects destructive follicular injury, for which thionamides are generally not useful. Finally, it underscores the value of individualized, case-based management guided by airway status and clinical response rather than radiological extent alone.

## 1 Introduction

Acute suppurative thyroiditis (AST) with abscess formation is one of the rarest endocrine emergencies of childhood. The thyroid gland resists infection through its fibrous capsule, abundant blood and lymphatic supply, and high iodine content. AST is rare but may progress to abscess formation and deep neck infection if diagnosis is delayed (1, 2). In children, the left lobe is disproportionately affected, a pattern linked to the pyriform sinus fistula (PSF), an embryological remnant of the third or fourth pharyngeal pouch that provides a conduit for oropharyngeal flora to reach the thyroid (3, 4). AST is easily mistaken for the far more common subacute thyroiditis, and a predisposing fistula is often sought only after recurrence (5). Deep neck space involvement adds diagnostic and airway concerns that further complicate management (6).

Although surgical drainage has traditionally been recommended for pediatric thyroid abscesses, emerging evidence suggests that selected patients may be managed successfully without surgery; the criteria for selecting these patients, however, remain poorly defined (2, 7). We report a child with a radiologically diagnosed left thyroid lobe abscess complicated by multispatial cervical extension and transient thyrotoxicosis, in whom no pyriform sinus fistula was demonstrated and who recovered without operative drainage following a non-yielding aspiration. Finally, we use this case to outline a practical framework for the surgery-versus-antibiotics decision.

## 2 Case description

A previously healthy 9-year-old boy presented with a 6-day history of high-grade fever (up to 39 °C) and sore throat radiating to the left ear, with odynophagia to solids and liquids developing on day 2 and hoarseness, neck stiffness, and pain on neck movement over the following 48 h. There was no stridor, drooling, respiratory distress, or symptoms of hyperthyroidism, and no prior thyroid disease, neck surgery, or recurrent neck infection. There was no family history of thyroid disease or congenital neck anomalies, and psychosocial and genetic history were non-contributory. During the prodrome, he had been assessed twice at other facilities and received analgesia only; no antibiotics were given before this presentation. On admission, he was febrile (38.7 °C), with a heart rate of 117 beats/min, blood pressure of 113/73 mmHg (mean arterial pressure of 86 mmHg), respiratory rate of 24 breaths/min, and oxygen saturation of 99% on room air. Examination revealed a patent airway, pharyngeal erythema, uvular deviation to the right, mild tenderness over the thyroid, and painful restriction of neck movement, but no external swelling, fluctuance, or clinical signs of thyrotoxicosis.

Laboratory tests showed a white cell count of 10.8 × 10^⁹^/L with neutrophil predominance, C-reactive protein (CRP) of 15.67 mg/dL (reference 0.10–0.50 mg/dL), and procalcitonin of 0.24 ng/mL (reference 0.02–0.10 ng/mL). Thyroid function testing revealed biochemical thyrotoxicosis: suppressed thyroid-stimulating hormone (TSH; 0.02 µIU/mL; normal 0.5–4.5), with elevated free T3 (7.48 pg/mL; normal 2.3–4.2) and free T4 (2.74 ng/dL; normal 0.8–1.8), consistent with acute thyroidal injury. Blood cultures showed no growth at 5 days, and respiratory viral PCR was negative.

A lateral neck radiograph showed prevertebral soft-tissue widening (Figure 1). Contrast-enhanced CT demonstrated a rim-enhancing collection within an enlarged left thyroid lobe (1.3 cm × 1.6 cm × 2.9 cm), extending into the visceral, prethyroid, and retropharyngeal spaces, and a contiguous smaller collection in the left parapharyngeal region (0.7 cm × 1.3 cm × 1.5 cm), with mass effect on the airway but no mediastinal extension or vascular thrombosis (Figures 2, 3). Flexible laryngoscopy showed a patent airway with normal, symmetrically mobile vocal cords and no pooling of secretions; no pyriform sinus fistula was demonstrated.

**Figure 1:**
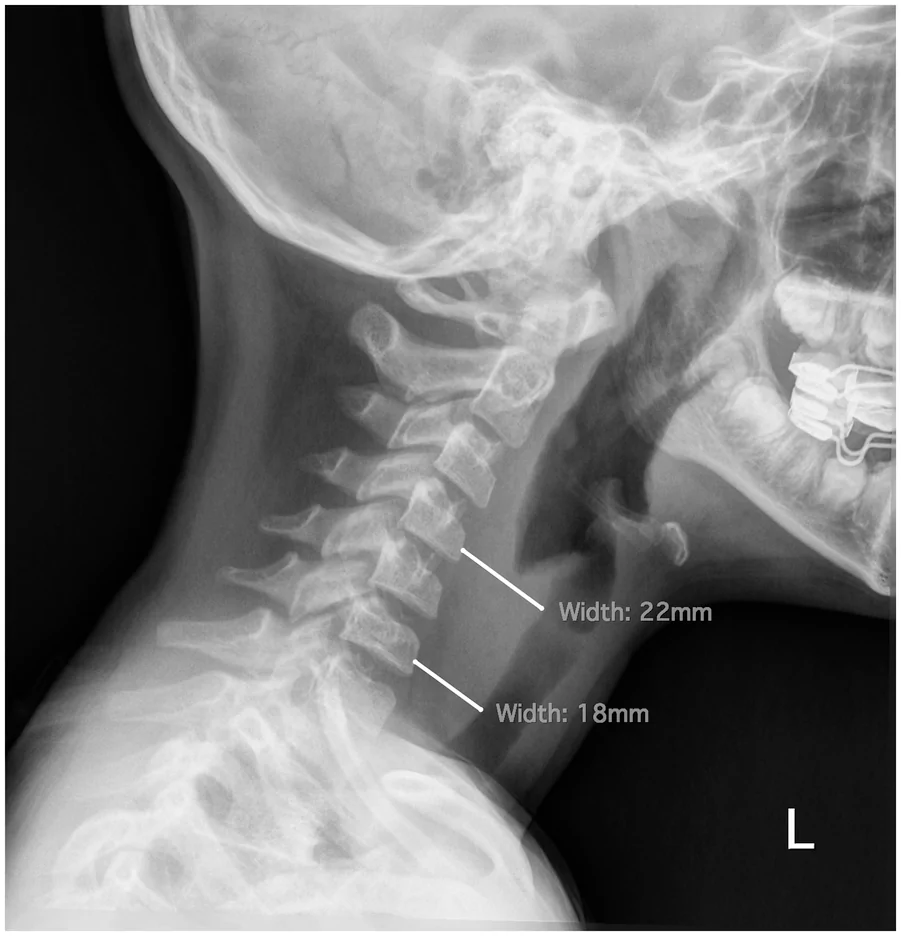
Lateral neck radiograph demonstrates a prevertebral soft-tissue widening measuring 22 mm at C4 and 18 mm at C6, suggestive of a deep neck space infection.

**Figure 2:**
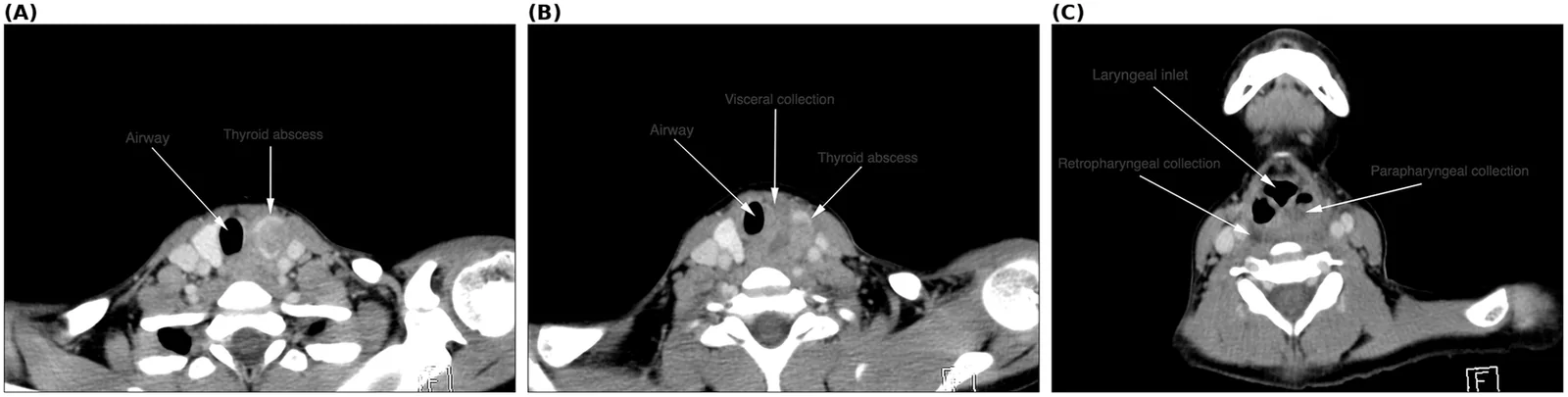
Contrast-enhanced axial CT images obtained at three levels demonstrate the multispatial extent of the infection. **(A)** A rim-enhancing hypodense collection within the left thyroid lobe (thyroid abscess) is shown; the airway is labeled. **(B)** The contiguous extension of the infection into the visceral space is shown; the airway and thyroid abscess are labeled. **(C)** In a more cranial view, the retropharyngeal extension with a small associated fluid collection in the left parapharyngeal region is shown; the laryngeal inlet is labeled.

**Figure 3:**
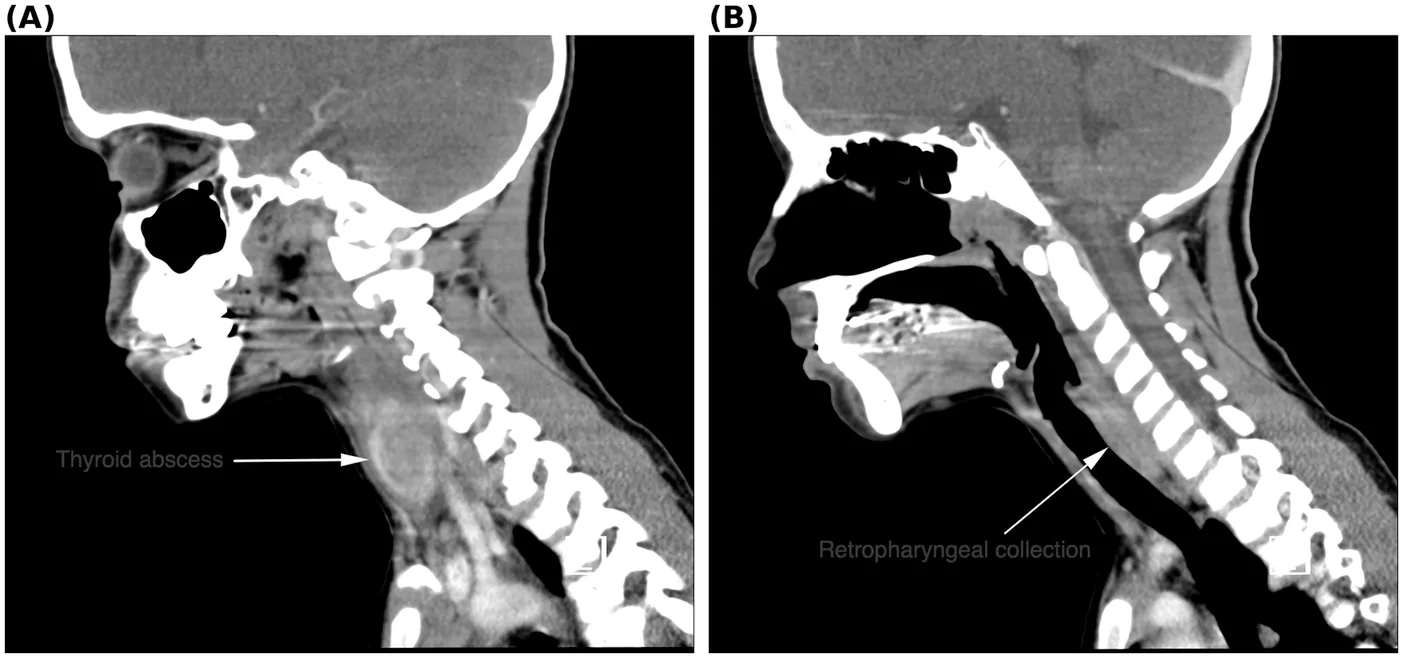
Contrast-enhanced sagittal CT images obtained at two sagittal levels demonstrate the craniocaudal extent of the infection. **(A)** The craniocaudal extent of the thyroid abscess (arrow) is shown. **(B)** The retropharyngeal extension along the prevertebral soft tissues (arrow) is shown.

Following a multidisciplinary discussion between the pediatric and otorhinolaryngology teams, conservative management was selected. Empiric intravenous ceftriaxone and clindamycin were started, and surgical drainage was not pursued. The patient was monitored with serial airway assessments, vital signs, inflammatory markers, and follow-up imaging; drainage or airway intervention would have been undertaken in the event of airway compromise, clinical deterioration or failure of inflammatory markers to improve within 48–72 h, abscess enlargement on repeat imaging, or systemic toxicity, none of which occurred.

The child became afebrile by day 2 with restored oral intake. CRP fell from 15.67 to 3.84 mg/dL and procalcitonin from 0.24 to 0.06 ng/mL by day 3. On day 4, under real-time ultrasound guidance, aspiration was attempted; the lesion appeared heterogeneous and predominantly hypoechoic with internal echoes rather than a discrete anechoic fluid collection. The aspiration needle tip was visualized within the target lesion throughout the procedure; however, no aspirable material was obtained for cytological or microbiological analysis. Together, the sonographic appearance and the non-yielding aspiration suggested limited liquefaction of the inflammatory collection. The diagnosis therefore remained based on imaging and clinical presentation, with subacute thyroiditis and an evolving inflammatory phlegmon without frank suppuration considered in the differential. On day 5, follow-up ultrasound demonstrated that the left thyroid-lobe collection measured 1.0 cm × 1.2 cm × 3.0 cm. Given the differences in imaging modality and imaging plane between the initial contrast-enhanced CT and the follow-up ultrasound, these findings were interpreted as showing no convincing interval progression rather than a measurable reduction. By day 7, CRP had normalized (0.54 mg/dL). The biochemical thyrotoxicosis was asymptomatic, so no antithyroid therapy was administered. Intravenous ceftriaxone and clindamycin were administered for 7 days, followed by oral cefixime and clindamycin for a further 3 weeks (total duration, 4 weeks); the oral combination was selected to maintain streptococcal, staphylococcal, and anaerobic coverage while allowing outpatient completion. The prescribed course was completed without reported adverse effects or tolerability issues.

The family did not attend the early scheduled follow-up visits and returned for follow-up approximately 14 months later. At that assessment, the child was clinically well with no reported recurrence. Thyroid function had normalized (TSH 2.32 µIU/mL, free T3 3.44 pg/mL, and free T4 0.98 ng/dL), and a barium swallow showed no radiological evidence of a pyriform sinus fistula. A repeat ultrasound was not performed as the child remained clinically well. The clinical course is summarized in Table 1.

**Table 1:** Summary of the clinical course.

Timepoint	Clinical/laboratory events	Interventions/decisions
Days −6 to −1	Fever, sore throat, evolving odynophagia, hoarseness, neck stiffness; seen twice elsewhere	Analgesia only; no antibiotics
Day 0 (admission)	Febrile 38.7 °C; uvular deviation; WBC 10.8; CRP 15.67; PCT 0.24; TSH 0.02, fT3 7.48, and fT4 2.74	Neck X-ray, contrast CT, laryngoscopy (no PSF); blood cultures; IV ceftriaxone+clindamycin
Day 2	Afebrile; oral intake restored; airway stable	Continued IV antibiotics
Day 3	CRP 3.84; PCT 0.06	Regimen unchanged
Day 4	Continued improvement	Ultrasound-guided aspiration attempted → non-yielding; no drainage
Day 5	Ultrasound: no interval progression in collection size (1.0 cm × 1.2 cm × 3.0 cm)	Conservative management confirmed
Day 7 (discharge)	CRP 0.54; asymptomatic; blood cultures negative at 5 days	Oral cefixime + clindamycin continued for 3 weeks (total antibiotic duration, 4 weeks)
Follow-up	Asymptomatic, no recurrence; TSH 2.32, fT3 3.44, and fT4 0.98 (normalized)	Barium swallow → no PSF

WBC, white cell count (×10^⁹^/L); CRP, C-reactive protein (mg/dL); PCT, procalcitonin (ng/mL); TSH, thyroid-stimulating hormone (µIU/mL); fT3, free triiodothyronine (pg/mL); fT4, free thyroxine (ng/dL); IV, intravenous; PSF, pyriform sinus fistula.

## 3 Discussion

This case illustrates that conservative management may succeed even in pediatric thyroid abscess with radiological multispatial extension when clinical stability is maintained. Three aspects of this case merit emphasis. The abscess extended into the visceral, prethyroid, retropharyngeal, and parapharyngeal spaces, an appearance that may ordinarily prompt drainage. The child also had biochemical thyrotoxicosis at presentation, an infrequent accompaniment of AST. He nonetheless recovered without operative drainage after a non-yielding aspiration.

Neither flexible laryngoscopy during the acute episode nor a barium swallow at follow-up demonstrated a pyriform sinus fistula, a commonly reported predisposing lesion in children (3, 4, 8). A single negative study does not, however, exclude an intermittently patent or collapsed tract, and re-investigation would be warranted should infection recur.

### 3.1 Clinical decision-making

The central question was whether radiological multispatial extension mandated drainage. Because the airway remained patent with no clinical signs of airway obstruction, systemic toxicity was absent, and imaging showed no mediastinal extension or vascular thrombosis, conservative treatment was considered appropriate. The child improved within 48 h, documented by serial reductions in CRP and procalcitonin. Together, these findings provided a practical rationale for conservative management, consistent with published pediatric and adult experience favoring individualized management of acute suppurative thyroiditis (7, 9). The empiric combination of ceftriaxone and clindamycin provided guideline-consistent broad-spectrum coverage for the pathogens commonly associated with acute suppurative thyroiditis (10). Given the multispatial involvement and ongoing clinical improvement, antibiotic therapy was continued for 4 weeks. These observations are hypothesis-generating and suggest that multispatial extension alone should not necessarily be considered an absolute indication for surgical drainage in carefully selected patients.

### 3.2 Transient thyrotoxicosis

Suppressed TSH with elevated free T3 and free T4 was consistent with destructive thyroiditis caused by follicular injury, not autonomous gland overactivity. The patient had no clinical thyrotoxicosis features. Because AST-associated thyrotoxicosis results from the destructive release of preformed thyroid hormone rather than increased hormone synthesis, thionamides are generally not useful, and management is supportive with monitoring of thyroid functions (11). Thyroid function was documented only at admission and at approximately 14 months, with no intermediate testing; the time course of recovery therefore cannot be characterized, and an intervening transient hypothyroid phase, which may follow destructive thyroiditis, cannot be excluded. Monitoring to confirm normalization and to detect later hypothyroidism remains appropriate.

### 3.3 Limitations

No causative organism was identified, as the aspiration was non-yielding and blood cultures were sterile. Follow-up was delayed and no outpatient ultrasound was obtained, so the precise timeline of biochemical and sonographic resolution is not documented; nonetheless, the child was asymptomatic with normalized thyroid function and no recurrence at reassessment. As with any single case, these findings should not be extrapolated to patients with airway compromise, systemic toxicity, mediastinal extension, or other features requiring urgent operative intervention. Nonetheless, because prospective data are unlikely for such a rare disease, carefully documented case reports remain valuable for refining patient selection for conservative management.

### 3.4 How this case differs from prior reports

Gaur et al. reported a pediatric thyroid abscess with transient hyperthyroidism managed successfully without operative drainage (12). The present case describes successful non-operative management of infection with multispatial extension beyond the thyroid after a non-yielding aspiration and without a demonstrable pyriform sinus fistula. Earlier pediatric reports have addressed conservative management (7), transient thyrotoxicosis (11), or pyriform sinus fistula (5, 8, 13) individually; the combination of multispatial extension and successful non-operative recovery is the principal distinguishing feature of this case.

In summary, conservative treatment may be feasible in carefully selected children with thyroid abscesses despite multispatial extension. Transient biochemical thyrotoxicosis accompanying acute suppurative thyroiditis reflects destructive thyroiditis, for which thionamides are generally not useful. Management should be guided primarily by airway status, systemic condition, and clinical response rather than CT appearance alone. Although prospective evidence is unlikely for such a rare condition, this case contributes to the limited evidence supporting an individualized management approach based on clinical stability rather than radiological extent alone.

## 4 Patient perspective

The patient's mother provided the following reflection: “My son became very unwell very quickly—he had a high fever and could barely swallow. When the doctors explained that there was an abscess in his thyroid gland, we were very frightened, especially when surgery was mentioned as a possibility. We felt reassured when the team decided to treat him with antibiotics first, and we could see him getting better day by day. By the time he was discharged after a week, he was eating normally and had returned to his usual self. We are grateful for the care he received, and he has remained well since.”

This perspective was provided verbally by the patient's mother and transcribed with her consent.

## Data Availability

The original contributions presented in the study are included in the article/Supplementary Material; further inquiries can be directed to the corresponding author.
